# Expression of Renal Vitamin D and Phosphatonin-Related Genes in a Sheep Model of Osteoporosis

**DOI:** 10.3390/ani12010067

**Published:** 2021-12-29

**Authors:** Keren E. Dittmer, Anastasia Chernyavtseva, Jonathan C. Marshall, Diana Cabrera, Frances M. Wolber, Marlena Kruger

**Affiliations:** 1 School of Veterinary Science, Massey University, Palmerston North 4442, New Zealand; anastasia.chernyavtseva@mpi.govt.nz; 2 School of Fundamental Sciences, Massey University, Palmerston North 4442, New Zealand; j.c.marshall@massey.ac.nz; 3 School of Food and Advanced Technology, Massey University, Palmerston North 4442, New Zealand; diana.cabrera@agresearch.co.nz (D.C.); F.M.Wolber@massey.ac.nz (F.M.W.); 4 School of Health Sciences, Massey University, Palmerston North 4442, New Zealand; M.C.Kruger@massey.ac.nz

**Keywords:** osteoporosis, sheep, vitamin D, phosphatonin, qPCR, calcium metabolism, phosphorus metabolism, *klotho*

## Abstract

**Simple Summary:**

Osteoporosis is a significant public health issue around the world, with post-menopausal osteoporosis due to estrogen deficiency resulting in approximately ¾ of cases. Treatment with glucocorticoids is another common cause of osteoporosis in humans. Sheep are a well-established model for osteoporosis in humans. In this study, aged sheep had their ovaries removed (ovariectomy) to simulate estrogen deficiency, and some sheep were also treated with glucocorticoids. The results showed that expression of the gene klotho in the kidney had the most marked difference in ovariectomized sheep treated with glucocorticoids for 2 months followed by a recovery period of 3 months. Klotho is known as the “anti-aging” hormone and is an important regulator of calcium and phosphorus metabolism. It may therefore be involved in the recovery of bone mineral density seen in ovariectomized sheep treated with glucocorticoids for 2 months followed by euthanasia at 5 months. As such, it could be an important treatment target for osteoporosis in humans.

**Abstract:**

Osteoporosis is a significant public health issue around the world, with post-menopausal osteoporosis due to estrogen deficiency resulting in approximately ¾ of cases. In this study, 18 aged Merino ewes were ovariectomized, and 10 were controls. Three of the ovariectomized ewes were treated weekly with 400 mg of methylprednisolone for 5 months and three were treated weekly for 2 months, followed by a 3-month recovery period. At 2 months, five control animals and six ovariectomized animals were euthanized. At 5 months, all the remaining ewes were euthanized. Kidney samples were collected postmortem for qPCR analysis of *NPT1*, *PTH1R*, *NPT2a*, *NPT2c*, *Klotho*, *FGFR1IIIc*, *VDR*, *CYP24A1*, *CYP27B1*, *TRPV5*, *TRPV6*, *CalD9k*, *CalD28k*, *PMCA* and *NCX1.* Ovariectomized sheep had significantly greater *VDR* expression compared with other groups. Ovariectomized sheep treated with glucocorticoids for 2 months followed by euthanasia at 5 months showed significant differences in *TRPV5*, *CYP24A1* and *klotho* gene expression compared to other groups. Differences in *klotho* expression were most marked after adjustment for repeated measures (*p* = 0.1). *Klotho* is known as the “anti-aging” hormone and is involved in calcium and phosphorus metabolism. *Klotho* may be involved in the recovery of bone mineral density in ovariectomized sheep treated with glucocorticoids for 2 months followed by euthanasia at 5 months. Further research on the role of *klotho* is recommended.

## 1. Introduction

Osteoporosis is a major public health issue for the world’s increasingly aging population, costing the United States of America nearly USD 17 billion in 2005 and projected to grow to USD 25 billion in 2025 [1,2]. Approximately 70–75% of the osteoporosis burden is due to post-menopausal osteoporosis in women [1], a primary osteoporosis whereby estrogen deficiency results in decreased bone formation and increased bone resorption, leading to decreased bone mass and bone fragility [3]. Osteoporosis may also occur secondary to specific causes, such as glucocorticoid treatment.

The sheep is a well-established large animal model for different human skeletal diseases, including osteoporosis [4]. One of the advantages of a large animal model, such as the sheep, is the ability to test orthopedic implants, surgical techniques and biomaterials [4]. Additionally, the sheep is relatively low cost, easy to handle and provides abundant material for testing [4,5].

The most common sheep models of osteoporosis are the 12 month post-operatively ovariectomized sheep and the 6 month ovariectomized sheep on a calcium and vitamin D deficient diet given glucocorticoids [4]. By using a combination of known osteoporosis-inducing factors including ovariectomy, glucocorticoid treatment and a low calcium diet, we have been able to establish a sheep model of osteoporosis in just 5 months [6,7], thus decreasing the housing, labor, and feed costs involved in model development. In this model, ovariectomy was found to increase the serum concentrations of C-terminal telopeptides of type I collagen (CTX-I, a bone resorption marker) and osteocalcin (bone turnover marker) compared with control sheep. Sheep that received glucocorticoid treatment for 2 months had higher serum CTX-I concentrations and lower serum osteocalcin concentrations. Additionally, femoral and lumbar spine bone density, and total and trabecular volumetric bone mineral density of the proximal tibia were lower in experimentally treated groups compared with the control groups, particularly for ovariectomized glucocorticoid treated ewes [6]. Amino acid and lipid metabolism was also found to be altered in ovariectomized and glucocorticoid treated sheep [7]. Similar changes have also been found in other ovine osteoporosis models [8].

Vitamin D responsive genes and the phosphatonin system have not previously been explored in ovariectomized sheep models. Vitamin D, of which 1,25-dihydroxyvitamin D is the active form, ultimately leads to increased plasma ionized calcium and phosphorus concentrations by binding to the vitamin D receptor (VDR)–retinoid X receptor heterodimer and altering expression of numerous vitamin D-responsive genes, such as calcium binding proteins (calbindin D9 and 28k (*CalD9k*, *CalD28k*)) and calcium channels (transient receptor potential cation channel subfamily V member 5 and 6 (*TRPV5*, *TRPV6*), plasma membrane calcium ATPase (*PMCA*), sodium calcium exchanger 1 (*NCX1*) [9]. The production of 1,25-dihydroxyvitamin D is a tightly regulated step, whereby the renal 1α hydroxylase (*CYP27B1*) catalyzes the conversion of 25-hydroxyvitamin D to 1,25-dihydroxyvitamin D, and the enzyme 24-hydroxylase (*CYP24A1*) breaks down both 25-hydroxyvitamin D and 1,25-dihydroxyvitamin D into inactive byproducts. The phosphatonin system also controls plasma phosphorus and calcium concentrations [10]. The lynch pin in this system is fibroblast growth factor 23, which is produced by osteocytes in bone and primarily controls plasma phosphorus concentrations by binding to the co-factor klotho to decrease the expression of sodium-phosphate co-transporters (*NPT1*, *NPT2a*, *NPT2c*) [11]. Due to the inter-related effects of parathyroid hormone, and 1,25-dihydroxyvitamin D, determining the effects of FGF23 on calcium metabolism has been challenging, but there is evidence to suggest it can regulate intestinal absorption of calcium and renal calcium reabsorption [12,13].

Glucocorticoids have numerous effects on calcium and phosphorus metabolism, including inhibiting intestinal calcium absorption, decreased renal tubular calcium reabsorption, and decreased renal phosphorus reabsorption [14,15,16]. Additionally, little research has examined the effect of estrogen deficiency on vitamin D-responsive genes and the phosphatonin system [17,18].

This ovariectomized, low calcium diet, glucocorticoid-treated sheep model of osteoporosis provided an opportunity to produce preliminary information on the effects of these treatments on the expression of phosphatonin and vitamin D-responsive genes in the ovine kidney. The aims of this study were twofold: (1) determine if ovariectomy or ovariectomy and glucocorticoid treatment altered the expression of vitamin D and phosphatonin-related genes in the kidney; (2) determine the relationships between renal vitamin D and phosphatonin-related genes, serum vitamin D and serum bone turnover markers (CTX-I and osteocalcin).

## 2. Materials and Methods

Detailed methods for the development of the ovariectomized and glucocorticoid-treated sheep model of osteoporosis have previously been published [6]. Briefly, aged Merino ewes (7–9 years old, *n* = 28) were randomly allocated into groups: control (*n* = 10), ovariectomized (OVX) (*n* = 12), ovariectomized and glucocorticoid treatment (400 mg methylprednisolone by subcutaneous injection) monthly for 2 months followed by no treatment for 3 months (*n* = 3) and glucocorticoid treatment (400 mg methylprednisolone) monthly for 5 months (*n* = 3). The sheep were housed in a barn and fed either a control (11.5 g/kg calcium; 2.2 g/sheep per day) or low calcium (5 g/kg calcium; 1 g/sheep per day) sheep pellet concentrate diet [19]. Maintenance requirements for a non-pregnant ewe are 1.2 g/sheep per day [19].

Blood samples were collected via jugular venipuncture 2 months and 5 months after surgery. Osteocalcin was measured using the MicroVue Osteocalcin immunoassay kit, and CTX-I using the IDS Serum CrossLaps ELISA kit, as previously reported [6]. Serum total calcium and phosphorus concentrations were measured using spectrophotometry on an AU680 Clinical Chemistry Analyzer (Beckman Coulter, Brea, CA, USA). Serum 25-hydroxyvitamin D concentration was measured using isotope dilution liquid chromatography mass spectrometry at the Endolab, Canterbury Health Laboratories, Christchurch, New Zealand.

At 2 months, 5 ewes from the control group and 6 from the OVX group were euthanized and necropsied. At 5 months, the remaining ewes were also euthanized (control group *n* = 5, OVX group *n* = 6, OVX plus 2 m glucocorticoids *n* = 3 and OVX plus 5 m glucocorticoids *n* = 5) and necropsied. Kidney samples were collected within 30 min of death and divided into two, with one sample placed in 10% neutral buffered formalin and processed for histology, and the other snap frozen in liquid nitrogen and stored at −80 °C until processing. Hematoxylin and eosin-stained sections of kidney were examined by the primary author and confirmed the absence of significant lesions.

All experimental procedures were approved by the Massey University Animal Ethics committee (approval number 14/103) and performed according to the Code of Ethical Conduct for the use of live animals for research at Massey University, Palmerston North, New Zealand.

RNA extraction and qPCR were performed as previously described [20,21]. Briefly, Tri Reagent (Sigma-Aldrich Inc., Merck KGaA, Darmstadt, Germany) was used to extract RNA as per the manufacturer’s instructions. Genomic DNA was removed with Ambion Turbo DNA free (Thermo Fisher Scientific Inc., Waltham, MA, USA) as per the manufacturer’s instructions. RNA concentration was determined using a Qubit 2.0 fluorometer and Qubit RNA broad range assay kit (Thermo Fisher Scientific Inc., Waltham, MA, USA), and RNA quality was assessed by determining the 260/280 ratio and running on an agarose gel. The Transcriptor first strand cDNA synthesis kit (Roche) was used to synthesize cDNA. Each 20 μL reaction mix contained 600 ng of RNA, 2.5 μM oligo(dT), 8 mM RT reaction buffer, 1 mM dNTP, 10 U reverse transcriptase, 20 U Rnase inhibitor and Rnase-Dnase-free water. The reaction was performed at 55 °C for 30 min, 85 °C for 5 min, and then chilled at 4 °C using an Applied Biosystems Veriti Thermal Cycler (Thermo Fisher Scientific Inc., Waltham, MA, USA). Real time qPCR was performed using the StepOne Plus real-time PCR machine (Applied Biosystems, Thermo Fisher Scientific Inc., Waltham, MA, USA). Each PCR mix contained 5 μL Power SYBR Green PCR master mix (Thermo Fisher Scientific Inc., Waltham, MA, USA), forward and reverse primers at the concentrations listed in Table S1, 10 ng of cDNA, and then made up to 10 μL with RNase, DNase-free water. The PCR protocol consisted of 95 °C for 20 s, 40 cycles at 95 °C for 3 s and 60 °C for 30 s and, finally, a melt curve ranging from 60 °C to 95 °C with a heating rate of 0.3 °C/15 s. Water and reaction mix without reverse transcriptase were included as negative controls in every PCR run, and all samples were run in duplicate. The following genes were assessed: *NPT1*, *PTH1R*, *NPT2a*, *NPT2c*, *Klotho*, *FGFR1IIIc*, *VDR*, *CYP24A1*, *CYP27B1*, *TRPV5*, *TRPV6*, *CalD9k*, *CalD28k*, *PMCA* and *NCX1.*

*PKG1* and *SDHA* have previously been found to be stably expressed in ovine kidney [20], and samples were normalized relative to the expression of these genes and gene efficiency using the 2^−ΔΔCt^ method [22].

Statistical analysis was performed using R Studio 1.3.1093 [23] with R version 4.0.3 and the tidyverse, nlme 3.1–150 and GGally 2.1.2 packages. Linear models were applied to the log10 transformed gene fold expression data. In the first model, gene expression was the outcome variable, while group (control or OVX but excluding glucocorticoid treated sheep) and time (euthanized at 2 m or 5 m) were dependent variables, with an interaction term (group:time) included. In the second model, gene expression was the outcome variable, while group (control, OVX, OVX with glucocorticoid treatment for 2 m, and OVX with glucocorticoid treatment for 5 m) was the dependent variable. As multiple outcome variables (genes) were being tested for differences between groups, *p*-values were adjusted for multiple testing using the sequential Holm procedure [24]. Serum calcium, phosphorus and 25-hydroxyvitamin D concentrations were tested in the gene expression models, but were not significant and, given the lack of significant differences between groups, were removed. Additionally, principal component analysis was used to assess if log10 transformed gene fold expression data differed by group, time of euthanasia, or serum 25-hydroxyvitamin D concentration. Spearman’s pairwise correlation and scatterplots were used to study the correlation between log10 transformed gene fold expression data and serum analytes.

## 3. Results

### 3.1. Effect of Ovariectomy, Time since Ovariectomy and Glucocorticoid Treatment on Serum Calcium, Phosphorus and 25-Hydroxyvitamin D Concentrations

**Figure 1:**
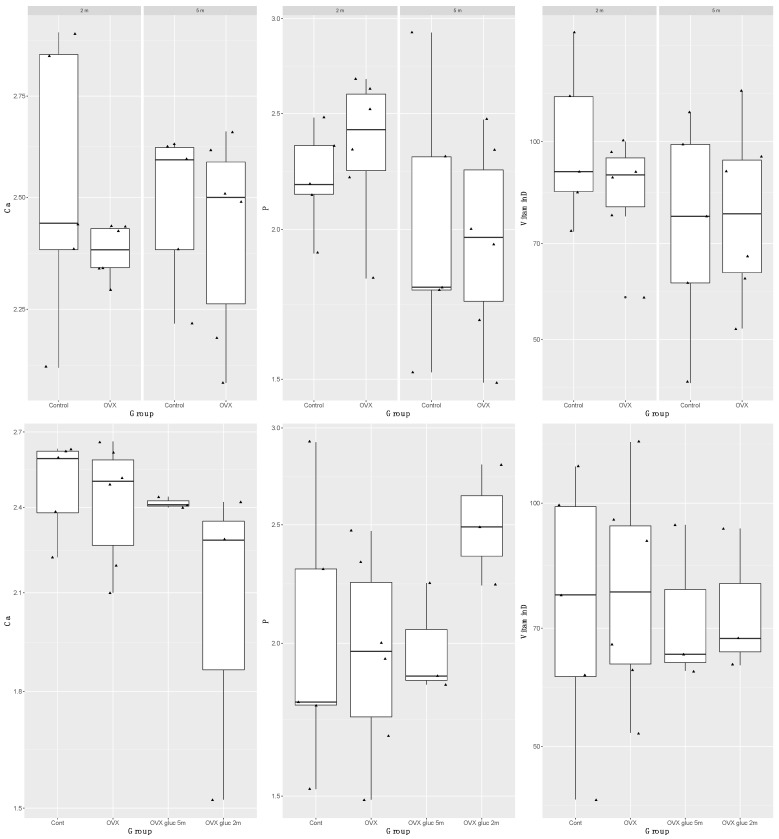
Boxplots of serum calcium (Ca), phosphorus (P) and 25-hydroxyvitamin D (Vitamin D) concentrations in control and ovariectomized (OVX) sheep euthanized at 2 months and 5 months of the trial (top line of boxplots), and in control (Cont), ovariectomized (OVX), OVX and glucocorticoids for 5 months, and OVX and glucocorticoids for 2 months followed by 3 months recovery, with euthanasia at 5 months.

The full results for serum calcium, phosphorus and 25-hydroxyvitamin D concentrations in each group are presented in Figure 1. There were no significant differences in serum calcium, phosphorus and 25-hydroxyvitamin D concentrations between the ovariectomized and low calcium diet group and the non-ovariectomized adequate calcium diet group, and time since ovariectomy of 2 months and 5 months. These results suggest ovariectomy combined with a low calcium diet had no impact on serum calcium concentrations. Sheep that were ovariectomized and treated with glucocorticoids for 2 months, followed by euthanasia at 5 months, had significantly lower serum calcium concentrations (*p* = 0.0462, adj R^2^ 0.13) compared with 5 m ovariectomized on a low calcium diet and 5 m ovariectomized on a low calcium diet and glucocorticoid-treated sheep. However, examination of the data suggests this may be due to one animal that was hypocalcemic (1.52 mmol/L, reference range 2.0–2.7 mmol/L); removal of this animal removed any significance. All other sheep had serum calcium concentrations within the reference range. No differences were seen in serum phosphorus or 25-hydroxyvitamin D concentrations in 5 m ovariectomized and glucocorticoid-treated sheep.

### 3.2. Effect of Ovariectomy and Time since Ovariectomy on Vitamin D and Phosphatonin-Related Gene Expression in the Kidney

**Figure 2:**
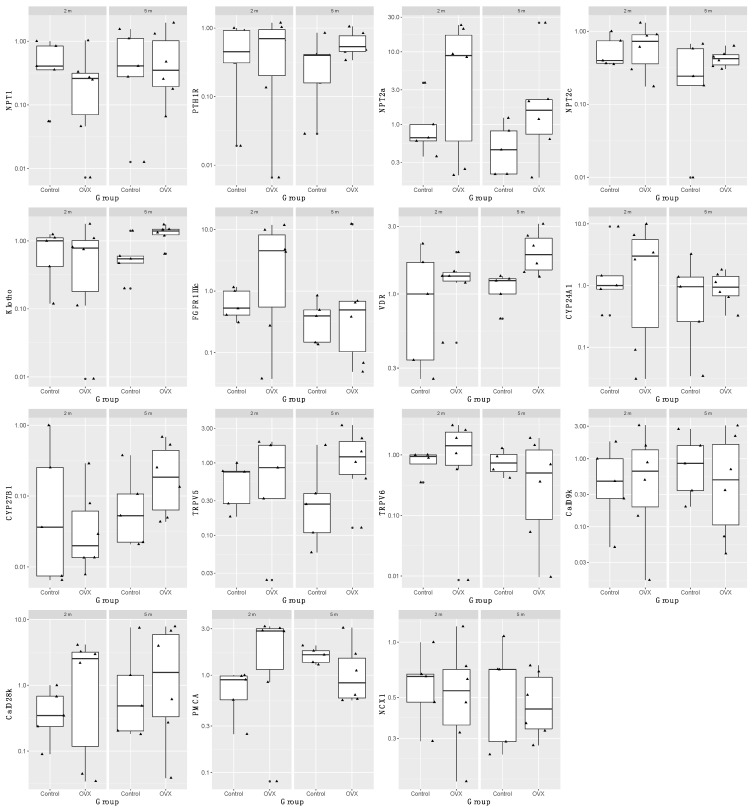
Boxplot of log_10_ transformed renal gene fold expression in control and ovariectomized (OVX) sheep euthanized at 2 and 5 months. Genes examined include: sodium-phosphate co-transporters (*NPT1*, *NPT2a*, *NPT2c*), parathyroid hormone 1 receptor (*PTH1R*), α-klotho, fibroblast growth factor receptor 1 IIIc (*FGFR1IIIc*), vitamin D receptor (*VDR*), cytochrome P450 family 24 subfamily A polypeptide 1 (*CYP24A1*), cytochrome P450 family 27 subfamily B polypeptide 1 (*CYP27B1*), transient receptor potential cation channel subfamily V member 5 and 6 (*TRPV5*, *TRPV6*), calbindin D9k (CalD9k), calbindin D28k (*CalD28k*), plasma membrane calcium ATPase (*PMCA*), sodium calcium exchanger 1 (NCX1). Black circle next to triangle indicates that point is an outlier.

Gene expression of vitamin D and phosphatonin-related genes was not associated with ovariectomy or time since ovariectomy (Figure 2). The exception was the vitamin D receptor (VDR), whereby the OVX group had significantly greater VDR expression (*p* = 0.05, adj R^2^ 0.16); however, this significance disappeared after adjustment for repeated measures.

### 3.3. Effect of Ovariectomy and Glucocorticoid Treatment on Vitamin D and Phosphatonin-Related Gene Expression in the KIDNEY 5 Months Post-Operatively

**Figure 3:**
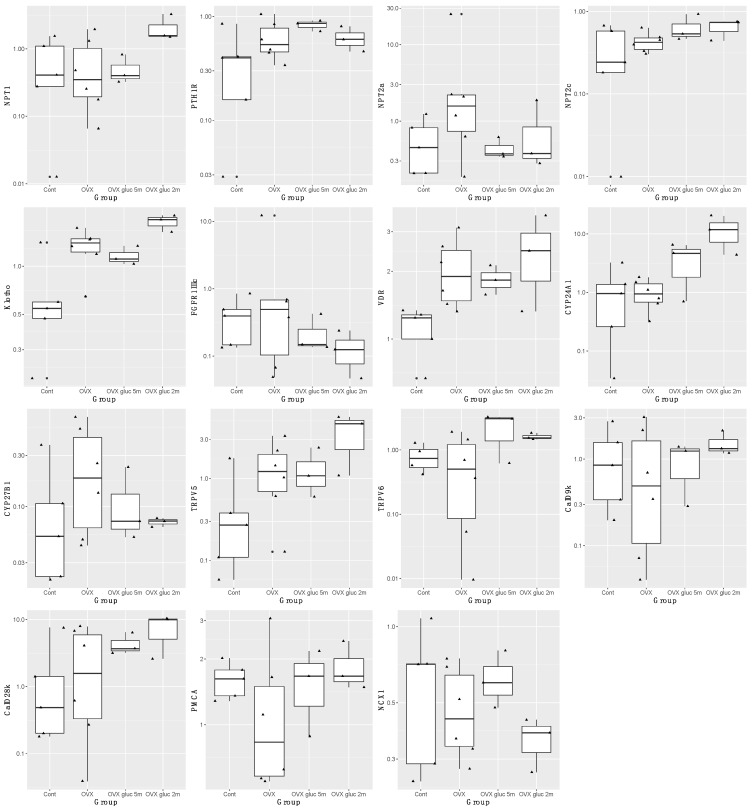
Boxplot of log_10_ transformed renal gene fold expression in control (Cont), ovariectomized (OVX), ovariectomized and treated with glucocorticoids for 5 months (OVX gluc 5m) and ovariectomized and treated with glucocorticoids for 2 months with 3 months recovery (OVX gluc 2m), all euthanized at 5 months. Genes examined include sodium-phosphate co-transporters (*NPT1*, *NPT2a*, *NPT2c*), parathyroid hormone 1 receptor (*PTH1R*), α-klotho, fibroblast growth factor receptor 1 IIIc (*FGFR1IIIc*), vitamin D receptor (*VDR*), cytochrome P450 family 24 subfamily A polypeptide 1 (*CYP24A1*), cytochrome P450 family 27 subfamily B polypeptide 1 (*CYP27B1*), transient receptor potential cation channel subfamily V member 5 and 6 (*TRPV5*, *TRPV6*), calbindin D9k (*CalD9k*), calbindin D28k (*CalD28k*), plasma membrane calcium ATPase (*PMCA*), sodium calcium exchanger 1 (*NCX1*). Black circle next to triangle indicates that point is an outlier.

The full results are presented in Figure 3. Expression of PTH1R was significantly greater in OVX sheep treated with glucocorticoids for 5 months compared with the other groups (*p* = 0.04, adj R^2^ 0.18), while TRPV5 (*p* = 0.01, adj R^2^ 0.31) and CYP24A1 (*p* = 0.005, adj R^2^ 0.40) expression was significantly greater in OVX sheep treated with glucocorticoids for 2 months compared with the other groups.

Expression of klotho was significantly different between all groups (*p* = 0.01, adj R^2^ 0.48), with the lowest expression in control sheep. OVX sheep treated with glucocorticoids for 2 months had the highest expression (*p* = 0.002), followed by OVX sheep (*p* = 0.008) and then OVX sheep treated with glucocorticoids for 5 months (*p* = 0.03). However, when adjusted for repeated measures, gene expression was not significantly different between these groups, with klotho showing the largest difference in OVX sheep treated with glucocorticoids for 5 months (*p* = 0.1).

Similarly, expression of the VDR was significantly different between all groups (*p* = 0.03, adj R^2^ 0.38). The greatest expression of the VDR was in OVX sheep treated with glucocorticoids for 2 months (*p* = 0.009), followed by OVX sheep (*p* = 0.01), then OVX sheep treated with glucocorticoids for 5 months (*p* = 0.05), and the lowest expression was present in control sheep.

### 3.4. PCA of Overall Gene Expression between Groups

PCA analysis of OVX, control and euthanasia at 2 and 5 months failed to separate out the different groups (Figure S1). PC1 and PC2 explained 69.6% of the variation. Similarly, PCA analysis of groups euthanized at 5 months also failed to separate out the different groups. PC1 and PC2 explained 53.2% of the variation (Figure S2). PCA analysis showed that overall gene expression could not be explained by serum calcium, phosphorus or 25-hydroxyvitamin D concentrations (Figure S3).

### 3.5. Pairwise Correlation between all Analytes and Gene Expression

**Figure 4:**
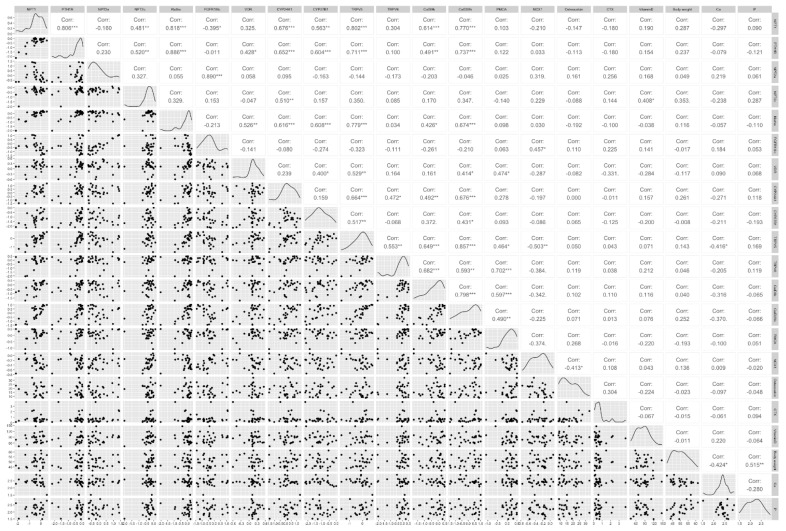
Pairwise Spearman’s correlation of phosphatonin and vitamin D-related transcripts (sodium-phosphate co-transporters (*NPT1*, *NPT2a*, *NPT2c*), parathyroid hormone 1 receptor (*PTH1R*), *α-klotho*, fibroblast growth factor receptor 1 IIIc (*FGFR1IIIc*), vitamin D receptor (*VDR*), cytochrome P450 family 24 subfamily A polypeptide 1 (*CYP24A1*), cytochrome P450 family 27 subfamily B polypeptide 1 (*CYP27B1*), transient receptor potential cation channel subfamily V member 5 and 6 (*TRPV5*, *TRPV6*), calbindin D9k (*CalD9k*), calbindin D28k (*CalD28k*), plasma membrane calcium ATPase (*PMCA*), sodium calcium exchanger 1 (*NCX1*)) in ovine kidneys, serum analytes (calcium (Ca), phosphorus (P), 25-hydroxyvitamin D (Vitamin D), osteocalcin, C-terminal telopeptide (CTX), and body weight. Bottom and left indicates log_10_ transformed renal gene fold expression. *** *p* < 0.001, ** *p* < 0.01, * *p* ≤ 0.05.

Regardless of ovariectomy status, calcium content of the diet, time since ovariectomy (2 m or 5 m) or glucocorticoid treatment status, *NPT1* gene expression was significantly and positively correlated (*p* < 0.001) with *PTH1R*, *NPT2c*, *Klotho*, *CYP24A1*, *TRPV5*, *CalD9k*, and *CalD28k* gene expression. *PTH1R* was significantly positively correlated (*p* < 0.001) with *NPT2c*, *Klotho*, *CYP24A1*, *CYP27B1*, *TRPV5*, and *CalD28k*. *NPT2a* was significantly positively correlated (*p* < 0.001) with *FGFR1IIIc*. *Klotho* was significantly positively correlated (*p* < 0.001) with *CYP24A1*, *CYP27B1*, *TRPV5* and *CalD28k. CYP24A1* was significantly positively correlated (*p* < 0.001) with *TRPV5* and *CalD28k. TRPV6* was significantly positively correlated (*p* < 0.001) with *Cal9Dk* and *PMCA*, while *Cal9DK* was significantly positively correlated (*p* < 0.001) with *CalD28k* and *PMCA*. None of the serum analytes measured (osteocalcin, CTX, 25OHD, Ca and P) were correlated with the expression of any genes. The full pairwise correlations are shown in Figure 4.

## 4. Discussion

The results of this study suggest that treatment of ovariectomized sheep with glucocorticoids has had the greatest impact on gene expression, while ovariectomy itself and time since ovariectomy (2 and 5 months) had little impact on gene expression. In a univariate analysis, significant differences in *TRPV5*, *CYP24A1* and *klotho* gene expression were seen in ovariectomized sheep treated with glucocorticoids for 2 months followed by euthanasia at 5 months, and in *PTH1R* in ovariectomized sheep treated with glucocorticoids for 5 months. Differences were most marked in *klotho* expression after adjustment for repeated measures.

Quantitative peripheral computed tomography of the proximal tibia of these sheep showed that ovariectomy decreased total volumetric bone mineral density (vBMD) by 8%, ovariectomy with glucocorticoid treatment for 5 months by 27%, and ovariectomy with glucocorticoids for 2 months and euthanasia at 5 months by 13% [6]. Interestingly, trabecular BMD, trabecular area and trabecular bone mineral content was similar or greater than controls in ovariectomized sheep treated with glucocorticoids for 2 months and euthanized at 5 months, suggesting recovery of bone mass [6].

Glucocorticoids have several well-documented effects on bone that lead to the development of glucocorticoid-induced osteoporosis. These effects include inhibiting the formation of osteoblast progenitor cells and osteoblasts, increasing osteoblast apoptosis, and decreasing osteoid production, all of which decrease bone formation [25,26]. At the same time, glucocorticoids increase bone resorption by decreasing OPG expression, increasing RANKL expression, increasing osteoclast formation, decreasing osteoclast apoptosis and increasing the secretion of cathepsins and matrix metalloproteinases by osteoclasts [25,26].

Klotho is an integral component of the phosphatonin system, whereby it binds to fibroblast growth factor 23 (FGF23) in the kidney. The klotho–FGF23 complex then binds to the FGFR1IIIC receptor, which initiates several cellular pathways and, ultimately, results in the upregulation of renal 24-hydroxylase (*CYP24A1*) expression and downregulation of renal 1a-hydroxyase (*CYP27B1*) and renal tubular Na/P II cotransporters (*NPT2a* and *2c*) [10]. The increased expression (differences in which were most marked after adjusting for repeated measures) of *klotho* in sheep on glucocorticoids for 2 months and then euthanized at 5 months may explain the increased *CYP24A1* expression also seen in this group of sheep, despite no significant differences in serum 25OHD concentrations.

Klotho is known as the anti-aging protein and *klotho*-deficient mice have low bone formation and bone resorption, leading to a low bone turnover osteopenia [27,28]. Additionally, specific *klotho* gene polymorphisms are associated with decreased bone mineral density in humans [29,30]. Soluble klotho upregulates early growth response protein 1 (EGR-1) and induces the expression of bone differentiation markers in osteoblast cell culture [31]. Additionally glucocorticoids can suppress fibroblast growth factor 23 [32]. Perhaps, the greater *klotho* expression in sheep on glucocorticoids for 2 months and then euthanized at 5 months is reflective of the recovery process for these sheep post-glucocorticoid treatment and, given its role in bone formation and resorption, conceivably a key driver of bone recovery and, therefore, potentially a treatment target for osteoporosis in humans.

However, OVX sheep also had increased *klotho* expression compared with control sheep. Similarly, one study in aromatase-deficient mice showed that estrogen deficiency increased klotho protein levels, and estradiol treatment decreased klotho expression in these mice [17]. In mice, estrogen downregulates *NPT2a* in a process that is independent of klotho/FGF23 and PTH [18]. However, this effect was not seen in the ovariectomized sheep in this present study. Otherwise, there is little literature on the effect of estrogen deficiency on klotho. Further research should examine the effects of both ovariectomy and glucocorticoids on *klotho* expression, given its role in aging and in chronic kidney disease.

*TRPV5* is an epithelial calcium channel chiefly expressed in the distal convoluted tubules and connecting tubules of the kidney [20,33]. The *TRPV5* channel is constitutively open, but undergoes calcium-dependent inactivation in association with calmodulin, a calcium-sensing protein [34]. *TRPV5* expression can be increased by PTH and 1,25(OH)_2_D_3_ [35]_._ One sheep that was on glucocorticoids for 2 months and then euthanized at 5 months was hypocalcemic, and serum calcium concentrations were significantly lower in sheep in this group compared with the other groups. It is likely, therefore, that the increased *TRPV5* expression is a result of the decreased serum calcium concentration in these animals. Glucocorticoids have been shown to decrease intestinal calcium absorption by decreasing expression of *TRPV6* and *CalD9k* [36,37] and increased urinary excretion of calcium [14], but clinically significant hypocalcemia in healthy individuals is uncommon. Similarly, the sheep with low serum calcium concentration was showing no clinical signs of hypocalcemic tetany. Alternatively, the increased expression of *TRPV5* in sheep on glucocorticoids for 2 months and then euthanized at 5 months could reflect the recovery process, whereby to replace the recovered trabecular BMD, area and bone mineral content, increased resorption of calcium from the kidney is required.

In sheep treated with glucocorticoids for 5 months, *PTH1R* expression was increased compared with the other groups. Additionally, compared to other groups, there was little variation in expression between sheep in the group. While no differences were seen in serum calcium concentrations in sheep treated with glucocorticoids for 5 months, perhaps this could be associated with the increased action of PTH and its receptor. The effects of glucocorticoids on PTH secretion and action are unclear. Some studies in humans have found that glucocorticoid-treated patients have increased serum PTH concentrations, along with decreased calcium absorption, increased urinary calcium excretion and decreased bone mass [38,39]. However, most studies have found no changes in serum PTH concentrations in humans on glucocorticoid treatment [38,40,41]. One study has shown that perhaps the impact of glucocorticoids is on altering the secretion of PTH, with increased pulsatile secretion of PTH in glucocorticoid-treated individuals [42]. Others have also shown increased *PTH1R* expression in mesenchymal stem cells and osteoblast-like cells in response to treatment with dexamethasone [43,44]. As such, the increase in renal *PTH1R* expression in the sheep treated with glucocorticoids for 5 months is likely due to the 5 months of glucocorticoid treatment.

While ovariectomy combined with a mildly low calcium diet resulted in minimal changes to gene expression, it did appear to alter expression of the *VDR*, with ovariectomized sheep on a mildly low calcium diet having greater *VDR* expression compared to control sheep. This is in contrast to a study on ovariectomized rats whereby those fed a diet with normal calcium levels showed increased renal *VDR* expression, while those fed a low calcium diet had decreased *VDR* expression [45]. It is difficult in this present sheep study to separate out the effect of ovariectomy on renal expression of the VDR from the effect of the low calcium diet. However, there were no significant differences in serum calcium concentrations between groups (with the exception of the glucocorticoid group), suggesting the mildly low calcium diet had minimal effect and perhaps the effects on the *VDR* are due to the ovariectomy. However, further research will be required to investigate these relationships.

Two previous studies have examined the separate relationships between vitamin D-responsive genes, and phosphatonin-related genes in sheep kidney [20,21]. However, relationships between the vitamin D and phosphatonin pathways have not been examined. Interestingly, most significant correlations between genes reported in these two papers hold true for the gene relationships in this present sheep study as well, suggesting consistency in the results between studies and the relationships between these genes. Of the interesting relationships not previously reported, as one might expect given its pivotal role in calcium metabolism, expression of *PTH1R* was highly correlated with *CalD28k*, *CalD9k*, *TRPV5*, *CYP27B1*, *CYP24A1*, *VDR*, *klotho* and *NPT2c*. Expression of *klotho* was strongly correlated with gene expression of *TRPV5*, which is consistent with other literature showing that klotho upregulates *TRPV5* and promotes renal calcium reabsorption [46,47].

Unfortunately, the different groups could not be separated based on vitamin D responsive and phosphatonin gene expression using PCA analysis. This is likely due to the main limitation of this study—the small sample size. With only 3–6 animals in each of the groups, this has likely limited the ability to detect differences in gene expression between groups.

## 5. Conclusions

The results from this study show that the most marked differences in gene expression were in renal klotho expression in ovariectomized sheep treated with glucocorticoids for 2 months followed by a recovery period of 5 months. Given klotho is the “anti-aging” hormone and has critical roles in calcium and phosphate metabolism, it is possible that this hormone is critically involved in the recovery of trabecular bone mineral density and area in treated sheep and may, therefore, potentially be a treatment target for osteoporosis in humans. However, more research is required on the effects of glucocorticoids and ovariectomy on klotho.

## Data Availability

Data will be available from the first author upon request.

## Supplementary Materials

The following supporting information can be downloaded at: https://www.mdpi.com/article/10.3390/ani12010067/s1, Figure S1 Principle component analysis of overall phosphatonin and vitamin D-related genes in ewes that had been ovariectomized for 2 months and 5 months, and control animals at 2 months and 5 months. Figure S2. Principle component analysis of overall phosphatonin and vitamin D-related genes in ewes that had been ovariectomized for 5 months, ovariectomized ewes treated with glucocorticoids for 5 months, ovariectomized ewes treated with glucocorticoids for 2 months, with 3 months recovery and control animals at 5 months. Figure S3. Principle component analysis of overall phosphatonin and vitamin D-related genes in ewes based on serum 25-hydroxyvitamin D concentrations. Table S1: Ovine phosphatonin and vitamin D-related kidney genes—transient receptor potential cation channel subfamily V member 5 (*TRPV5*), transient receptor potential cation channel subfamily V member 6 (*TRPV6*), Calbindin D9k (*calD9k*), Calbindin D28k (*calD28k*), plasma membrane calcium ATPase (*PMCA*), sodium calcium exchanger 1 (*NCX1*), cytochrome P450 family 27 subfamily B polypeptide 1 (*CYP27B1*), cytochrome P450 family 24 subfamily A polypeptide 1 (*CYP24A1*), vitamin D receptor (*VDR*), fibroblast growth factor receptor 1 IIIc (*FGFR1IIIc*), α-klotho (*klotho*), sodium-phosphate co-transporter 1 (*NPT1*), *NPT2a*, *NPT2c*, parathyroid hormone 1 receptor (*PTH1R*), succinate dehydrogenase complex (*SDH*) and phosphoglycerate kinase 1 (*PGK1*).

## References

[1] Burge R., Dawson-Hughes B., Solomon D.H., Wong J.B., King A., Tosteson A. (2007). Incidence and economic burden of osteoporosis-related fractures in the United States, 2005–2025. J. Bone Miner. Res..

[2] Camacho P.M., Petak S.M., Binkley N., Diab D.L., Eldeiry L.S., Farooki A., Harris S.T., Hurley D.L., Kelly J., Michael Lewiecki E. (2020). American association of clinical endocrinologists/American college of endocrinology clinical practice guidelines for the diagnosis and treatment of postmenopausal osteoporosis—2020 update. Endocr. Pract..

[3] Noh J.Y., Yang Y., Jung H. (2020). Molecular mechanisms and emerging therapeutics for osteoporosis. Int. J. Mol. Sci..

[4] Dias I.R., Camassa J.A., Bordelo J.A., Babo P.S., Viegas C.A., Dourado N., Reis R.L., Gomes M.E. (2018). Preclinical and translational studies in small ruminants (Sheep and Goat) as models for osteoporosis research. Curr. Osteoporos. Rep..

[5] Turner A.S. (2002). The sheep as a model for osteoporosis in humans. Vet. J..

[6] Cabrera D., Wolber F.M., Dittmer K., Rogers C., Ridler A., Aberdein D., Parkinson T., Chambers P., Fraser K., Roy N.C. (2018). Glucocorticoids affect bone mineral density and bone remodelling in OVX sheep: A pilot study. Bone Rep..

[7] Cabrera D., Kruger M., Wolber F.M., Roy N.C., Fraser K. (2020). Effects of short- and long-term glucocorticoid-induced osteoporosis on plasma metabolome and lipidome of ovariectomized sheep. BMC Musculoskelet. Disord..

[8] Coelho C.A., Bordelo J.P., Camassa J.A., Barros V.A., Babo P.S., Gomes M.E., Reis R.L., DE AZEVEDO J.T., Requicha J.F., Faísca P. (2020). Evaluation of hematology, general serum biochemistry, bone turnover markers and bone marrow cytology in a glucocorticoid treated ovariectomized sheep model for osteoporosis research. An. Acad. Bras. Cienc..

[9] Dittmer K.E., Thompson K.G. (2011). Vitamin D metabolism and rickets in domestic animals: A review. Vet. Pathol..

[10] Hardcastle M.R.R., Dittmer K.E.E. (2015). Fibroblast growth factor 23: A new dimension to diseases of calcium-phosphorus metabolism. Vet. Pathol..

[11] Wagner C., Hernando N., Forster I., Biber J. (2014). The SLC34 family of sodium-dependent phosphate transporters. Pflügers Arch.-Eur. J. Physiol..

[12] Khuituan P., Wongdee K., Jantarajit W., Suntornsaratoon P., Krishnamra N., Charoenphandhu N. (2013). Fibroblast growth factor-23 negates 1,25(OH)2D3-induced intestinal calcium transport by reducing the transcellular and paracellular calcium fluxes. Arch. Biochem. Biophys..

[13] Han X., Yang J., Li L., Huang J., King G., Quarles L.D. (2016). Conditional deletion of fgfr1 in the proximal and distal tubule identifies distinct roles in phosphate and calcium transport. PLoS ONE.

[14] Suzuki Y., Ichikawa Y., Saito E., Homma M. (1983). Importance of increased urinary calcium excretion in the development of secondary hyperparathyroidism of patients under glucocorticoid therapy. Metabolism.

[15] Cosman F., Nieves J., Herbert J., Shen V., Lindsay R. (1994). High-dose glucocorticoids in multiple sclerosis patients exert direct effects on the kidney and skeleton. J. Bone Miner. Res..

[16] Freiberg J.M., Kinsella J., Sacktor B. (1982). Glucocorticoids increase the Na+-H+ exchange and decrease the Na+ gradient-dependent phosphate-uptake systems in renal brush border membrane vesicles. Proc. Natl. Acad. Sci. USA.

[17] Öz O.K., Hajibeigi A., Howard K., Cummins C.L., Van Abel M., Bindels R.J.M., Word R.A., Kuro-o M., Pak C.Y.C., Zerwekh J.E. (2007). Aromatase deficiency causes altered expression of molecules critical for calcium reabsorption in the kidneys of female mice. J. Bone Miner. Res..

[18] Webster R., Sheriff S., Faroqui R., Siddiqui F., Hawse J.R., Amlal H. (2016). Klotho/fibroblast growth factor 23- and PTH-independent estrogen receptor-α-mediated direct downregulation of NaPi-IIa by estrogen in the mouse kidney. Am. J. Physiol.-Ren. Physiol..

[19] Sykes A.R., Grace N., Knowles S., Sykes A. (2010). Calcium. Managing Mineral Deficiencies in Grazing Livestock.

[20] Azarpeykan S., Dittmer K.E.K.E., Marshall J.C.J.C., Perera K.C.K.C., Gee E.K.E.K., Acke E., Thompson K.G.K.G. (2016). Evaluation and comparison of vitamin d responsive gene expression in ovine, canine and equine kidney. PLoS ONE.

[21] Dittmer K.E., Heathcott R.W., Marshall J.C., Azarpeykan S. (2020). Expression of phosphatonin-related genes in sheep, dog and horse kidneys using quantitative reverse transcriptase pcr. Animals.

[22] Pfaffl M.W. (2001). A new mathematical model for relative quantification in real-time RT–PCR. Nucleic Acids Res..

[23] R Studio Team (2020). RStudio: Integrated Development for R RStudio.

[24] Holm S. (1979). A simple sequentially rejective multiple test procedure. Scand. J. Stat..

[25] Adami G., Saag K.G. (2019). Glucocorticoid-induced osteoporosis: 2019 concise clinical review. Osteoporos. Int..

[26] Peng C.-H., Lin W.-Y., Yeh K.-T., Chen I.-H., Wu W.-T., Lin M.-D. (2021). The molecular etiology and treatment of glucocorticoid-induced osteoporosis. Tzu-Chi Med. J..

[27] Kuro-o M., Matsumura Y., Aizawa H., Kawaguchi H., Suga T., Utsugi T., Ohyama Y., Kurabayashi M., Kaname T., Kume E. (1997). Mutation of the mouse klotho gene leads to a syndrome resembling ageing. Nature.

[28] Torres P.U., Prié D., Molina-Blétry V., Beck L., Silve C., Friedlander G. (2007). Klotho: An antiaging protein involved in mineral and vitamin D metabolism. Kidney Int..

[29] Zarrabeitia M.T., Hernández J.L., Valero C., Zarrabeitia A.L., Ortiz F., Gonzalez-Macias J., Riancho J.A. (2007). Klotho gene polymorphism and male bone mass. Calcif. Tissue Int..

[30] Yamada Y., Ando F., Niino N., Shimokata H. (2005). Association of polymorphisms of the androgen receptor and klotho genes with bone mineral density in Japanese women. J. Mol. Med..

[31] Toan N.K., Tai N.C., Kim S.A., Ahn S.G. (2020). Soluble Klotho regulates bone differentiation by upregulating expression of the transcription factor EGR-1. FEBS Lett..

[32] Feger M., Ewendt F., Strotmann J., Schäffler H., Kempe-Teufel D., Glosse P., Stangl G.I., Föller M. (2021). Glucocorticoids dexamethasone and prednisolone suppress fibroblast growth factor 23 (FGF23). J. Mol. Med..

[33] Boros S., Bindels R., Hoenderop J. (2009). Active Ca2+ reabsorption in the connecting tubule. Pflugers Arch. Eur. J. Physiol..

[34] Dang S., van Goor M.K., Asarnow D., Wang Y., Julius D., Cheng Y., van der Wijst J. (2019). Structural insight into TRPV5 channel function and modulation. Proc. Natl. Acad. Sci. USA.

[35] van Goor M.K.C., Hoenderop J.G.J., van der Wijst J. (2017). TRP channels in calcium homeostasis: From hormonal control to structure-function relationship of TRPV5 and TRPV6. Biochim. Biophys. Acta-Mol. Cell Res..

[36] Hahn T.J., Halstead L.R., Baran D.T. (1981). Effects of short term glucocorticoid administration on intestinal calcium absorption and circulating vitamin d metabolite concentrations in man. J. Clin. Endocrinol. Metab..

[37] Huybers S., Naber T.H.J., Bindels R.J.M., Hoenderop J.G.J. (2007). Prednisolone-induced Ca^2+^ malabsorption is caused by diminished expression of the epithelial Ca^2+^ channel TRPV6. Am. J. Physiol.-Gastrointest. Liver Physiol..

[38] Rubin M.R., Bilezikian J.P. (2002). The role of parathyroid hormone in the pathogenesis of glucocorticoid-induced osteoporosis: A re-examination of the evidence. J. Clin. Endocrinol. Metab..

[39] Fucik R.F., Kukreja S.C., Hargis G.K., Bowser E.N., Henderson W.J., Williams G.A. (1975). Effect of glucocorticoids on function of the parathyroid glands in man. J. Clin. Endocrinol. Metab..

[40] Slovik D.M., Neer R.M., Ohman J.L., Lowell F.C., Clark M.B., Segre G.V., Potts J.T. (1980). Parathyroid hormone and 25-hydroxyvitamin D levels in glucocorticoid-treated patients. Clin. Endocrinol..

[41] Paz-Pacheco E., El-Hajj Fuleihan G., Leboff M.S. (1995). Intact parathyroid hormone levels are not elevated in glucocorticoid-treated subjects. J. Bone Miner. Res..

[42] Bonadonna S., Burattin A., Nuzzo M., Bugari G., Rosei E.A., Valle D., Iori N., Bilezikian J.P., Veldhuis J.D., Giustina A. (2005). Chronic glucocorticoid treatment alters spontaneous pulsatile parathyroid hormone secretory dynamics in human subjects. Eur. J. Endocrinol..

[43] Ahlström M., Pekkinen M., Lamberg-Allardt C. (2009). Dexamethasone downregulates the expression of parathyroid hormone-related protein (PTHrP) in mesenchymal stem cells. Steroids.

[44] Haramoto N., Kawane T., Horiuchi N. (2007). Upregulation of PTH receptor mRNA expression by dexamethasone in UMR-106 osteoblast-like cells. Oral Dis..

[45] Zhang Y., Lai W.P., Wu C.F., Favus M.J., Leung P.C., Wong M.S. (2007). Ovariectomy worsens secondary hyperparathyroidism in mature rats during low-Ca diet. Am. J. Physiol.-Endocrinol. Metab..

[46] Andrukhova O., Smorodchenko A., Egerbacher M., Streicher C., Zeitz U., Goetz R., Shalhoub V., Mohammadi M., Pohl E.E., Lanske B. (2014). FGF23 promotes renal calcium reabsorption through the TRPV5 channel. EMBO J..

[47] Wolf M.T.F., An S.W., Nie M., Bal M.S., Huang C.L. (2014). Klotho up-regulates renal calcium channel transient receptor potential vanilloid 5 (TRPV5) by intra- and extracellular n-glycosylation-dependent mechanisms. J. Biol. Chem..

