# Supplementary material for: Expression of Renal Vitamin D and Phosphatonin-Related Genes in a Sheep Model of Osteoporosis
Source: Animals (Basel). 2021 Dec 29;12(1):67. doi: 10.3390/ani12010067 (PMC8749731; doi:10.3390/ani12010067)
Supplement: Supplementary file 1 [file animals-12-00067-s001.zip › Table S1.pdf]

Supplementary Table S1. Ovine phosphatonin and vitamin D-related kidney genes - transient receptor potential cation channel subfamily V member 5 (TRPV5), transient receptor potential cation channel subfamily V member 6 (TRPV6), Calbindin $D_{9}k$ ($calD_{9k}$), Calbindin $D_{28}k$ ($calD_{28k}$), plasma membrane calcium ATPase (PMCA), sodium calcium exchanger 1 (NCX1), cytochrome P450 family 27 subfamily B polypeptide 1 (CYP27B1), cytochrome P450 family 24 subfamily A polypeptide 1 (CYP24A1), vitamin D receptor (VDR), fibroblast growth factor receptor 1 IIIc (FGFR1IIIc), α-klotho (klotho), sodium-phosphate co-transporter 1 (NPT1), NPT2a, NPT2c, parathyroid hormone 1 receptor (PTH1R), succinate dehydrogenase complex (SDH) and phosphoglycerate kinase 1 (PGK1). Primer sequences from Azarpeykan et al. 2016 and Dittmer et al. 2020[1,2]

Gene	Full gene name	Primer (5'-3')	Amplicon length (bp)	Primer concentration nM F:R	PCR Efficiency %	Regression coefficient (R^2)
**TRPV5**	Transient receptor potential cation channel subfamily V member 5	F: CGGGTCAGCAATCATCCTAT<br>R: ATTGTGATGACGTGGAATGG	108	250:250	110.6	0.96
**TRPV6**	Transient receptor potential cation channel subfamily V member 6	F: TGATGCTGGAGAAGAAGCTG<br>R: TGGTTGATGTCCTGTTTCTCTT	118	250:250	93.9	0.98
**$calD_{9k}$**	Calbindin D9k	F: TCACTGCTGAACGGCCAGGACA<br>R: AGCTCCTCCTTGGACAGTTGGT	122	250:250	99.3	0.99
**$calD_{28k}$**	Calbindin D28k	F: GCTGGAAAAAGCAAACAAGACTGTTGA<br>R: TTCTCCTGCACGGGTAGTAATCTGG	139	400:400	90.8	0.98
**PMCA**	Plasma membrane calcium ATPase	F: TGCAGCCATAGTATCATTGGGCCT<br>R: TTGCCGCTCCTTCAATCCAACCA	128	250:250	94.9	0.99
**NCX1**	Sodium calcium exchanger 1	F: TGGCGAACATCAACCCGTGCT<br>R: TGCAGATTGTAGCGTCGCATCTCG	93	300:300	98.4	0.99
**CYP27B1**	Cytochrome P450 family 27 subfamily B polypeptide 1	F: GCAGAGCTTGAGTTGCACAT<br>R: CTTCTCTCAGGCACCAGGAC	119	250:250	102.2	0.92
**CYP24A1**	Cytochrome P450 family 24 subfamily A polypeptide 1	F: CTGTGATGAGAGAGGCCGCATTGA<br>R: AGCTTCCTCCCCTGCCTTCTT	128	600:600	103.9	0.99
**VDR**	Vitamin D receptor	F: TCATGCTGCGCTCCAACCAGT<br>R: TGGAACTTGATGAGGGGCTCGAT	140	400:400	93.5	0.98
**FGFR1IIIc**	Fibroblast growth factor receptor 1 IIIc	F: ACACCACCGACAAAGAGATGG<br>R: TCAGCCATGCAGAGTGATGG	120	900:900	99.3	0.99

**Klotho**	Alpha-klotho	F: TGTGGAGAATGGCTGGTTTGT<br>R: TACCCAATGACATCCACCCC	129	300:300	96.0	1.00
**NPT1 (SLC17A1)**	Sodium-phosphate co-transporter 1	F: TCCCACTGGCAGCTGAATTT<br>R: CTCAGAGCAGTAAGTCGGCC	148	300:300	98.1	1.00
**NPT2a (SLC34A1)**	Sodium-phosphate co-transporter 2a	F: CACCATGACCCACTGCCTG<br>R: TCCAGGGAGCAGACGAAGAG	149	300:300	98.8	0.99
**NPT2c (SLC34A3)**	Sodium-phosphate co-transporter 2c	F: CGACATCCTCAAGGTGCTGA<br>R: AATAAGGCTCCGGTTGGTGG	109	300:300	100	0.99
**PTH1R**	Parathyroid hormone 1 receptor	F: CAGTACCGGAAGCTGCTCAA<br>R: TTGCCAGAGAGTCCCTGAGA	111	900:900	98.6	1.00
**SDHA**	Succinate dehydrogenase complex	F: ACCTGATGCTTTGTGCTCTGC<br>R: CCTGGATGGGCTTGGAGTAA	126	300:300	97.1	0.99
**PGK1**	Phosphoglycerate kinase 1	F: ACTCCTTGCAGCCAGTTGCT<br>R: AGCACAAGCCTTCTCCACTTCT	101	300:300	94.5	0.99

1. Azarpeykan S, Dittmer KEKE, Marshall JCJC, et al. Evaluation and Comparison of Vitamin D Responsive Gene Expression in Ovine, Canine and Equine Kidney. *PLoS One*. 2016;11:e0162598.
2. Dittmer KE, Heathcott RW, Marshall JC, Azarpeykan S. Expression of phosphatonin-related genes in sheep, dog and horse kidneys using quantitative reverse transcriptase pcr. 2020;10:1806.
